# Is Age-Related Hearing Loss a Modifiable Risk Factor for Cognitive Decline? Mechanisms, Evidence, and Future Directions

**DOI:** 10.3390/audiolres16020061

**Published:** 2026-04-21

**Authors:** Giovanni Motta, Giuseppe Tortoriello, Domenico Testa

**Affiliations:** 1 Otolaryngology Head and Neck Surgery Unit, Azienda Ospedaliera di Rilievo Nazionale dei Colli, Ospedale Monaldi, 80131 Naples, Italy; dott.giuseppetortoriello@virgilio.it; 2 ENT Unit, Department of Mental, Physical Health and Preventive Medicine, University of Campania “Luigi Vanvitelli”, 80131 Naples, Italy; domenico.testa@unicampania.it

**Keywords:** age-related hearing loss (ARHL), cochlear implants (CIs), hearing aids (HAs), cognitive decline, dementia risk, hearing rehabilitation, listening effort, sensory deprivation, cortical plasticity, social isolation, neurocognitive aging, modifiable risk factors

## Abstract

**Background**: Age-related hearing loss (ARHL) is the most common sensory disorder in older adults and has been identified as a potentially modifiable risk factor for cognitive decline and dementia. Increasing evidence suggests that auditory dysfunction may contribute to adverse cognitive trajectories through multiple interacting pathways. This narrative review examines the mechanisms underlying the association between ARHL and cognitive decline, evaluates the impact of hearing rehabilitation, and discusses future research priorities. **Methods**: A narrative synthesis of epidemiological, neurobiological, and interventional studies was conducted, with emphasis on longitudinal cohort studies, neuroimaging research, and clinical investigations of hearing aids (HAs) and cochlear implants (CIs). **Results**: ARHL is consistently associated with accelerated cognitive decline and increased dementia risk. Proposed mechanisms include sensory deprivation-related cortical reorganization, increased cognitive load during effortful listening, shared neuropathological processes, and social disengagement. Neuroimaging studies demonstrate structural and functional alterations in auditory and associative brain regions in individuals with hearing loss. Emerging evidence suggests that HA and CI may improve cognitive performance and potentially attenuate decline, although long-term randomized data remain limited. **Conclusions**: Current evidence supports ARHL as a clinically relevant and potentially modifiable contributor to cognitive decline. Clarifying causal pathways and optimizing early hearing rehabilitation strategies represent key priorities for future dementia prevention research.

## 1. Introduction

Age related hearing loss (ARHL), or presbycusis, is the most common sensory impairment in older adults and affects a substantial and growing proportion of the aging population worldwide. Prevalence increases steeply with advancing age, exceeding 60–70% among individuals over 70 years of age. Beyond its well-established impact on communication and quality of life, ARHL has increasingly been recognized as a condition with potential implications for broader brain health [1,2].

Over the past decade, epidemiological studies have consistently reported associations between ARHL and reduced cognitive performance, accelerated cognitive decline, and increased risk of incident dementia [1,2,3,4]. A large systematic review and meta-analysis demonstrated that ARHL is associated with poorer global cognition and executive function, as well as increased odds of cognitive impairment and dementia [3]. These findings have been replicated across multiple longitudinal cohort studies [1,2,4]. Furthermore, ARHL has been identified as one of the potentially modifiable risk factors for dementia in major prevention frameworks, including the influential report by Livingston et al., which highlighted hearing loss as a leading modifying risk factor in midlife [5,6].

While epidemiological associations are robust, the mechanisms linking ARHL and cognitive decline remain incompletely understood. Age-related auditory decline involves not only peripheral cochlear dysfunction but also widespread changes in central auditory pathways and cortical networks involved in speech perception and higher-order processing [7]. Neuroimaging studies have demonstrated structural and functional alterations in auditory and associative cortices in individuals with hearing loss, suggesting that ARHL may be accompanied by broader brain changes extending beyond primary sensory regions [7,8].

Several explanatory models have been proposed. The sensory deprivation hypothesis posits that chronic reduction in auditory input may lead to downstream neuroplastic and neurodegenerative changes in cortical networks [8]. The cognitive load hypothesis suggests that increased listening effort due to degraded auditory input may tax domain-general cognitive resources over time [8]. Alternative models emphasize shared etiological pathways, such as vascular pathology or generalized neurodegeneration, which may concurrently affect auditory and cognitive systems [8]. Additionally, social and behavioral consequences of hearing impairment, including reduced social engagement, may indirectly contribute to adverse cognitive trajectories [5,6].

Despite accumulating evidence, critical questions remain regarding causality, temporal dynamics, and the potential reversibility of risk. Observational studies suggest that hearing rehabilitation through HA or CI may be associated with improved cognitive outcomes [9,10], yet definitive randomized evidence is still emerging. Clarifying whether ARHL directly contributes to neurocognitive decline- or instead reflects parallel aging processes- has substantial implications for dementia prevention strategies [5,6].

In this review, we synthesize current epidemiological and mechanistic evidence linking ARHL and cognitive decline. We examine candidate biological and psychosocial pathways, evaluate interventional findings, and identify key gaps that must be addressed to determine whether ARHL represents a viable target for cognitive health preservation in aging populations.

## 2. Materials and Methods

This narrative review was designed to provide a comprehensive and integrated overview of current evidence on ARHL as a potentially modifiable risk factor for cognitive decline, with particular attention to underlying mechanisms, clinical interventions, and future research opportunities. While narrative in nature, the review employed a structured and rigorous methodology to ensure transparency, reproducibility, and scientific robustness.

A systematic and exhaustive search was conducted across Pubmed/MEDLINE, Scopus, and Web of Science from database inception through March 2026. Search strategies combined controlled vocabulary (MeSH terms) and free-text keywords, including: “age-related hearing loss”, “presbycusis”, “hearing impairment”, “cognitive decline”, “dementia”, “mild cognitive impairment”, “hearing aids”, “cochlear implants”, “neuroplasticity”, and “listening effort”. Boolean operators were used to refine sensitivity and specificity. Additionally, reference lists of landmark studies, recent meta-analyses, and narrative reviews were manually screened to capture pertinent literature not indexed in the primary search.

Studies were considered eligible if they included adult populations, primarily older individuals, with ARHL, and investigated at least one of the following domains: epidemiological associations between hearing loss and cognitive outcomes, neurobiological or mechanistic pathways linking auditory and cognitive function, or the impact of hearing rehabilitation strategies such as hearing aids or cochlear implants. Eligible studies were required to report cognitive outcomes, including global cognition, cognitive decline, mild cognitive impairment, or incident dementia, and to be published in peer-reviewed journals in English. Both longitudinal and cross-sectional observational studies, as well as interventional and neuroimaging investigations, were considered for inclusion.

Studies were excluded if they involved pediatric populations or congenital hearing loss, focused on non-age-related forms of hearing impairment (such as acute, traumatic, or infectious etiologies), or did not report relevant cognitive, mechanistic, or interventional outcomes. Additional exclusion criteria included case reports, editorials, letters, conference abstracts, and studies lacking sufficient methodological detail or clearly defined hearing assessment methods. Duplicate publications or studies with overlapping datasets were also excluded unless they provided additional relevant information.

**Figure 1:**
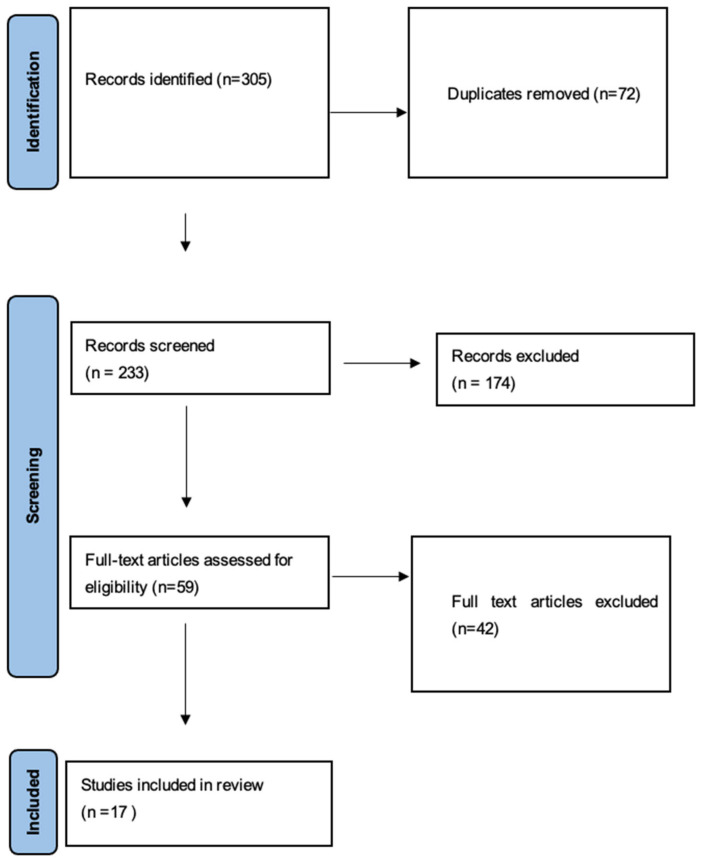
PRISMA-style flow diagram of study selection.

The study selection process followed a structured approach. A total of 305 records were initially identified, and after removal of duplicates, titles and abstracts were screened for relevance. Full-text articles were subsequently assessed according to the predefined eligibility criteria. Overall, 17 studies met the inclusion criteria and were included in the qualitative synthesis. The study selection process is summarized in a PRISMA-style flow diagram (Figure 1).

From each included study, key information was extracted, including study design, participant characteristics, methods used to assess hearing loss, cognitive outcomes, main findings, and reported limitations. This allowed a structured comparison across studies and facilitated thematic synthesis.

Given the heterogeneity in study designs, populations, and outcomes measures, a qualitative synthesis approach was adopted. The strength of evidence was assessed qualitatively, taking into account study design (with greater weight assigned to longitudinal and interventional studies), sample size, consistency of findings across studies, use of objective measures of hearing and cognition, and potential sources of bias, including confounding and reverse causation. The extracted evidence was then synthesized across major domains, including epidemiological associations, mechanistic pathways, interventional findings, and knowledge gaps.

## 3. Results

A total of 305 records were identified through database searching and additional sources. After removal of duplicates, 233 records were screened by title and abstract, of which 174 were excluded. 59 full-text articles were assessed for eligibility, and 17 studies were ultimately included in the qualitative synthesis (Figure 1).

The included studies comprised longitudinal cohort studies, cross-sectional analyses, neuroimaging investigations, and interventional studies evaluating hearing rehabilitation. The majority of studies involved older adult populations and assessed hearing loss using audiometric or self-reported measures, while cognitive outcomes included global cognition, executive function, memory, and incident dementia. The main characteristics of the included studies are summarized in Table 1.

Across the included longitudinal and observational studies, a consistent association between ARHL and adverse cognitive outcomes emerged. Prospective cohort studies demonstrated that individuals with hearing loss have an increased risk of dementia and accelerated cognitive decline over time, even after adjustment for demographic and vascular confounders [1,2,4]. In particular, a dose–response relationship was observed, with greater severity of hearing loss associated with progressively higher dementia risk [1]. Meta-analytic evidence confirmed these findings, showing significant associations between ARHL and reduced global cognition, executive dysfunction, and increased likelihood of mild cognitive impairment and dementia [3,11]. Despite the consistency of these findings, heterogeneity in cognitive assessment tools and potential residual confounding were noted across studies. Additionally, some studies raised the possibility of reverse causation, although the longitudinal design of several cohorts supports a temporal relationship in which hearing loss precedes cognitive decline [1,2,4].

Neuroimaging and experimental studies included in this review provided converging evidence supporting multiple mechanistic pathways linking ARHL and cognitive decline. Structural imaging studies demonstrated reduced gray matter volume in primary auditory cortex, temporal regions, and frontal associative areas in individuals with hearing loss [7,8,12,13]. Functional imaging studies further showed altered connectivity within language and executive networks, suggesting widespread neural reorganization. These findings support the sensory deprivation hypothesis, according to which reduced auditory input leads to decreased cortical stimulation and subsequent neuroplastic changes [8,12]. In parallel, experimental studies showed increased frontal activation during speech processing tasks in individuals with hearing impairment, consistent with compensatory recruitment of executive resources [14]. This pattern aligns with the cognitive load hypothesis, which proposes that effortful listening imposes sustained demands on domain-general cognitive systems. In addition, several studies highlighted the potential role of shared neuropathological processes, including vascular dysfunction and neurodegeneration, which may simultaneously affect auditory and cognitive systems [5,6,12]. Psychosocial pathways were also consistently reported, with social isolation and reduced engagement identified as mediating factors linking hearing impairment to cognitive decline [5,6].

Evidence from interventional and longitudinal observational studies suggests that hearing rehabilitation may influence cognitive outcomes. Studies evaluating hearing aid use consistently reported associations with slower cognitive decline compared to untreated hearing loss [9,15]. Some neuroimaging investigations also suggested partial normalization of cortical activity following auditory rehabilitation [13,15]. Cochlear implantation studies provided additional evidence, with prospective investigations demonstrating improvements in executive function, working memory, and global cognition in older adults following implantation [10,16]. However, the strength of this evidence is limited by heterogeneity in study design, variability in adherence, and the predominance of observational data. Randomized controlled evidence remains limited, although recent findings indicate that hearing intervention may reduce cognitive decline in specific high-risk subgroups, highlighting the importance of population characteristics in determining treatment effects [17].

Overall, the included studies consistently support an association between ARHL and cognitive decline across epidemiological, neurobiological, and interventional domains. While the strength of evidence varies across domains, converging findings suggest that multiple interacting mechanisms may underlie this relationship, although definitive causal inference remains limited.

A summary of the main domains, key findings, and limitations is presented in Table 2.

## 4. Discussion

The findings of the present review, as summarized in Section 3 and Table 2, demonstrate a consistent association between ARHL and cognitive decline across epidemiological, neurobiological, and interventional domains. Importantly, the strength of this association does not rely on a single line of evidence but emerges from the convergence of longitudinal cohort studies, meta-analyses, mechanistic investigations, and early interventional data.

As outlined in Section 3, epidemiological evidence represents the most robust and consistent domain. Longitudinal cohort studies included in this review consistently show that hearing loss is associated with an increased risk of incident dementia and accelerated cognitive decline, even after adjustment for major confounders [1,2,4]. The seminal work by Lin et al. demonstrated not only this association but also a dose–response relationship, whereby the hazard ratio for dementia increased progressively with the severity of hearing impairment [1]. This gradient, replicated across subsequent studies, strengthens the plausibility of a meaningful relationship rather than a spurious association. Meta-analytic findings further reinforce these observations, confirming significant associations between ARHL and global cognition, executive function, and dementia risk across heterogeneous populations [3,11].

However, as previously highlighted in Section 3, epidemiological findings must be interpreted with caution. Residual confounding cannot be entirely excluded, as factors such as socioeconomic status, education, and healthcare access may influence both hearing outcomes and cognitive trajectories. Additionally, reverse causation remains a theoretical concern, since early neurodegenerative changes could affect central auditory processing prior to overt cognitive decline [12]. Nevertheless, the temporal sequence observed in longitudinal studies- where baseline hearing loss predicts subsequent cognitive deterioration- argues against reverse causation as the sole explanation [1,2,4].

Mechanistic evidence provides biological plausibility for these epidemiological associations. Structural neuroimaging studies included in this review consistently demonstrate reduced gray matter volume in auditory and associative cortical regions among individuals with ARHL [7,8,13]. These findings support the sensory deprivation hypothesis, according to which reduced auditory input leads to decreased cortical stimulation and subsequent neuroplastic changes [8,12]. In parallel, functional studies reveal altered activation patterns, particularly increased recruitment of frontal regions during speech processing tasks, reflecting compensatory neural mechanisms [13,14].

The cognitive load hypothesis offers a complementary explanation for these findings. As described, experimental studies indicate that degraded auditory input increases listening effort, leading to sustained engagement of executive control systems [14]. Over time, this increased cognitive demand may reduce the availability of resources for other cognitive processes, such as memory encoding and executive functioning. Importantly, sensory deprivation and cognitive load mechanisms are not mutually exclusive; rather, they likely interact, with structural and functional changes jointly contributing to cognitive vulnerability.

In addition to these mechanisms, Section 3 also highlight the potential role of shared neuropathological processes and psychosocial pathways. Vascular dysfunction and neurodegenerative processes may simultaneously affect auditory and cognitive systems, complicating causal interpretation [5,6,12]. Furthermore, hearing loss is frequently associated with social isolation and reduced engagement, both of which are established risk factors for cognitive decline [5,6]. These findings suggest that ARHL may influence cognition through both direct neurobiological effects and indirect behavioral pathways.

The interventional evidence provides important, albeit still limited, insights into potential reversibility. Observational studies consistently report that hearing aid use is associated with slower cognitive decline [9,15], while cochlear implantation studies demonstrate improvements in executive function and global cognition in older adults [10,16]. These findings suggest that restoring auditory input may have beneficial effects on cognitive function, possibly through reactivation of cortical networks or reduction in cognitive load. However, the predominance of observational designs, variability in adherence, and limited follow-up duration restrict the strength of causal inference.

Recent randomized evidence, including the ACHIEVE trial, further refines this picture. While no significant effect was observed in the overall population, subgroup analyses demonstrated a beneficial impact of hearing intervention in individuals at higher risk of cognitive decline [17]. This finding suggests that the effect of hearing rehabilitation may not be uniform but instead depends on baseline risk profiles and population characteristics.

Taken together, the evidence synthesized in this review supports the conceptualization of ARHL as a clinically relevant and potentially modifiable contributor to cognitive decline. However, the heterogeneity of study designs and the limitations identified across epidemiological and interventional domains indicate that causality cannot yet be definitively established. Future research should therefore prioritize large-scale randomized controlled trials, longitudinal neuroimaging studies assessing structural and functional reversibility, and integrated approaches incorporating vascular, neurodegenerative, and behavioral factors.

From a clinical perspective, these findings have important implications. Given the high prevalence of ARHL and its potential contribution to cognitive decline, early identification and management of hearing loss should be considered an integral component of geriatric assessment. Routine audiological screening in midlife and older adults may allow timely intervention, potentially mitigating downstream cognitive consequences [5,6]. Furthermore, integrating hearing rehabilitation into multidisciplinary dementia prevention strategies could represent a pragmatic and scalable approach to reducing population-level-risk.

In conclusion, the present review highlights that the relationship between ARHL and cognitive decline is multifactorial, involving interacting biological and psychosocial mechanisms. While current evidence strongly supports an association, further high-quality studies are required to determine the extent to which hearing rehabilitation can effectively modify long-term cognitive trajectories.

Future research should aim to better disentangle causal pathways through well-designed longitudinal studies, incorporating multimodal biomarkers, including neuroimaging, genetic, and vascular markers. In addition, randomized controlled trials with longer follow-up durations are needed. Particular attention should be paid to identifying subpopulations that may derive the greatest benefit from intervention, as suggested by recent evidence [17].

## 5. Conclusions

This narrative review demonstrates that ARHL is consistently associated with cognitive decline and increased dementia risk, supported by epidemiological, mechanistic, and interventional evidence. Multiple pathways, including sensory deprivation, increased cognitive load, shared neuropathology, and psychosocial factors, likely contribute to this relationship.

While hearing rehabilitation shows promise as a potential intervention, current evidence remains insufficient to establish a definitive causal effect on long-term cognitive outcomes. Further well-designed longitudinal and randomized studies are required to determine whether targeting hearing loss can effectively reduce cognitive decline and dementia risk.

## Figures and Tables

**Table 1:** Main characteristics of the included studies examining the associations between ARHL and cognitive decline.

Study	Design	Population	Hearing Assessment	Cognitive Outcomes	Key Findings	Limitations
Frank R. Lin et al., 2011 [1]	Longitudinal cohort	Older adults	Pure-tone audiometry	Incident dementia	Dose–response association between HL severity and dementia risk	Residual confounding
Frank R. Lin et al., 2013 [2]	Longitudinal cohort	Older adults	Audiometry	Global cognition	Accelerated cognitive decline in individuals with HL	Reverse causation possible
Deal et al., 2017 [4]	Longitudinal cohort	Community-dwelling older adults	Audiometry	Dementia, cognition	Increased dementia risk in HL	Attrition bias
Darragh G. Loughrey et al., 2018 [3]	Systematic review and meta-analysis	Mixed populations	Mixed	Global cognition, dementia	Significant association between HL and cognitive impairment	Heterogeneity across studies
Ford et al., 2018 [11]	Longitudinal cohort	Older adults	Mixed	Dementia	HL associated with increased dementia risk	Measurement variability
Katherine Slade et al., 2020 [7]	Narrative review	NA	NA	Brain structure/function	HL linked to brain structural and functional changes	Narrative design
Ian S. Johnsrude and Wayne, 2015 [8]	Review	NA	NA	Mechanisms	Sensory deprivation and cognitive load hypotheses	Conceptual framework
Uchida et al., 2019 [12]	Review	NA	NA	Mechanisms	Multifactorial mechanisms linking HL and cognition	Limited causal evidence
Gill Livingston et al., 2017 [5]	Commission report	Population-level	NA	Dementia risk	HL identified as major modifiable risk factor	Not primary data
Gill Livingston et al., 2024 [6]	Commission report	Population-level	NA	Dementia risk	HL confirmed as leading modifiable risk	Indirect evidence
Peelle et al., 2011 [13]	Neuroimaging (cross-sectional)	Older adults	Audiometry	Speech comprehension	Altered cortical activation in HL	Small sample size
Mary K. Pichora-Fuller et al., 2016 [14]	Experimental	Adults	Audiometry	Listening effort	Increased cognitive load in HL	Laboratory setting
Dawes et al., 2015 [15]	Longitudinal cohort	Adults	Audiometry	Cognitive decline	HL associated with longitudinal decline	Confounding variables
Maharani et al., 2018 [9]	Longitudinal cohort	Older adults	Self-report	Cognitive function	HA use associated with slower decline	Self-selection bias
Mosnier et al., 2015 [10]	Interventional (CI)	Elderly patients	Audiometry	Cognition	Significant cognitive improvement post-CI	No control group
Cosetti et al., 2016 [16]	Interventional (CI)	Older adults	Audiometry	Neurocognition	Improved executive and global cognition	Small sample size
Peelle et al., 2011 (functional analysis) [13]	Neuroimaging	Older adults	Audiometry	Neural networks	Reduced efficiency in speech processing networks	Cross-sectional design

**Table 2:** Summary of the main domains linking ARHL and cognitive decline, including key findings, representative evidence, qualitative strength of evidence, and main limitations. Strength of evidence was qualitatively assessed considering consistency of findings across studies, study design, and overall methodological quality.

Domain	Key Findings	Representative References	Strength of Evidence	Limitations
Epidemiological Association	ARHL associated with increased risk of cognitive decline and dementia; dose–response relationship observed	[1,2,3,4,11]	Strong and consistent	Residual confounding; reverse causation possible
Sensory Deprivation Mechanism	Cortical atrophy and reduced neural stimulation in auditory and associative regions	[7,8,13]	Moderate to strong	Longitudinal causal inference limited
Cognitive Load Mechanism	Increased listening effort and frontal compensatory activation	[8,14]	Moderate	Long-term impact inferred from short-term paradigms
Shared Neuropathology	Vascular and neurodegenerative overlap between auditory and cognitive decline	[5,6,12]	Moderate	Mechanistic disentanglement challenging
Psychosocial Pathways	Social isolation mediates cognitive vulnerability	[5,6]	Moderate	Mediation models heterogeneous
Hearing Aids (HA)	HA use associated with slower cognitive decline and possible cortical normalization	[9,13,15]	Moderate	Observational bias; adherence variability
Cochlear Implants (CI)	CI associated with improvements in executive and global cognition	[10,16]	Moderate to strong (short–mid term)	Limited RCTs; small samples in some cohorts

## Data Availability

The original contributions presented in this study are included in the article. Further inquiries can be directed to the corresponding author.

## Abbreviations

The following abbreviations are used in this manuscript:

ARHL	Age- related hearing loss
CI	Cochlear implant
HA	Hearing aid
